# Exploring metabolomic clues in diabetic retinopathy: a pilot study

**DOI:** 10.1007/s00592-026-02678-5

**Published:** 2026-03-17

**Authors:** Matthew Simonson, Yanliang Li, J. Jason McAnany, Jason C. Park, Felix Y. Chau, Bharati Prasad, Silvana Pannain, Erin C. Hanlon, Eve Van Cauter, Kirstie K. Danielson, Brian T. Layden, Hui Chen, George E. Chlipala, Carlos Martinez, Stephanie J. Crowley, Sirimon Reutrakul

**Affiliations:** 1 College of Medicine, University of Illinois Chicago, Chicago, IL 60612 USA; 2 Department of Ophthalmology and Visual Sciences, University of Illinois, Chicago, Chicago, IL USA; 3 Division of Pulmonary, Critical Care, Sleep and Allergy, Department of Medicine, University of Illinois Chicago, Chicago, IL USA; 4 Jesse Brown Department of Veterans Affairs Hospital, Chicago, IL USA; 5 Section of Adult and Pediatric Endocrinology, Diabetes and Metabolism, Department of Medicine, University of Chicago, Chicago, IL USA; 6 Division of Endocrinology, Diabetes and Metabolism, Department of Medicine, University of Illinois Chicago, Chicago, IL USA; 7 Mass Spectrometry Core, Research Resource Center, Office of Vice Chancellor for Research, University of Illinois at Chicago, Chicago, IL USA; 8 Research Informatics Core, Research Resources Center, University of Illinois at Chicago, Chicago, IL USA; 9 Biological Rhythms Research Laboratory, Department of Psychiatry & Behavioral Sciences, Rush University Medical Center, Chicago, IL USA

**Keywords:** Metabolomics, Diabetic retinopathy, Diabetes Mellitus, Retina

## Introduction

Diabetic retinopathy (DR) is one of the most common microvascular complications of diabetes and a primary cause of blindness among working-age adults [1]. In 2020, 103.12 million individuals lived with DR worldwide, corresponding to a global prevalence of 22.27% among people with diabetes [1]. Prevalence is expected to increase; by 2045, an estimated 160.50 million adults worldwide will be afflicted by DR, and 44.82 million will experience vision-threatening DR [1]. 

Early detection is necessary for reducing DR progression. Despite major risk factors such as hyperglycemia, hypertension, and dyslipidemia, considerable variability in DR onset and progression remains unexplained. This suggests a need to explore pathophysiological mechanisms and biomarker identification. Emerging evidence from metabolomics—the study of small-molecule metabolites in biological systems—has shown promise in uncovering molecular alterations associated with diabetic complications, including DR [2]. Previous studies identified metabolites like 12-hydroxyeicosatetraenoic acid (12-HETE) and 3,4-dihydroxybutyrate (3,4-DHBA) as DR-related [2], pointing to inflammation and altered lipid metabolism. However, few studies include healthy controls (HC) in addition to DR and no-DR groups [2]. Recognizing the need for more comprehensive designs, this study uses untargeted metabolomics to examine DR, no-DR, and HC groups to identify biomarkers and gain insight into DR pathogenesis.

## Materials and methods

We analyzed baseline fasting serum samples from 77 participants aged 40–65 years, drawn from a cohort who had previously enrolled in sleep and circadian rhythm studies conducted at a tertiary academic hospital. *Post-illumination pupillary light reflex (PIPR)*,* a measure of intrinsically photosensitive retinal ganglion cells (ipRGCs) located in the inner retina*,* and nocturnal urinary 6-sulfatoxymelatonin (aMT6s); both previously shown to be reduced in DR*,* were collected using standardized protocols*,* previously described.* [3] Participants included 26 T2D without DR (no-DR), 36 T2D with moderate-severe nonproliferative DR, and 15 HCs. DR severity was graded per the Early Treatment Diabetic Retinopathy Study classification [4]. Inclusion required a recent ophthalmologic evaluation. Key exclusion criteria included ocular disease unrelated to diabetes, systemic conditions affecting the retina, and recent melatonin or illicit drug use. Obstructive sleep apnea, known to be associated with DR [5], was assessed using the apnea-hypopnea index (AHI) via an overnight home diagnostic device, the WatchPAT 200/300^®^.

Blood samples were collected following an overnight fast and assayed for hemoglobin A1C (HbA1c), lipids and serum creatinine (Quest Diagnostics), and serum was stored at -80 °C until analyzed. Metabolomic profiling was conducted using an untargeted LC-MS (Agilent 6545 Q-TOF, 1290 UPLC system). Raw data files were processed using the Profinder software (vB.10.00, Agilent Technologies). Features were extracted and filtered based on quality metrics and retention time. Metabolite data were normalized and analyzed using the *limma* package with empirical Bayes adjustment, including covariates (age, sex, BMI, blood pressure, LDL, triglycerides, serum creatinine, AHI, smoking status, medication use). *Exploratory correlations between serum metabolites and urinary aMT6s and PIPR were assessed using biweight midcorrelation while controlling for age*,* sex*,* BMI*,* and pAHI.* Adjusted p-values (q-values) were calculated using the Benjamini–Hochberg false discovery rate correction. Log-transformed, scaled data were subjected to principal component analysis (PCA) in R to explore global metabolic differences, with plots displaying the first two components for DR, no-DR, and HCs. *Pathway enrichment between DR and no-DR was explored using Ingenuity Pathway Analysis (IPA; Qiagen). Data are presented as mean (SD)*,* median (IQR)*,* or frequency (%)*,* and group comparisons were performed using Wilcoxon rank-sum*,* Welch’s t-test*,* or Chi-square as appropriate.*

## Results

Table 1 shows participant demographics, which were comparable across DR and no-DR groups in age, sex, BMI, and lipid levels. The DR group had longer diabetes duration and greater insulin use than the no-DR group, consistent with disease severity. Systolic blood pressure was higher in DR participants *while urinary aMT6s and PIPR were lower*. HCs demonstrated less medication use, lower triglycerides, blood pressure, HbA1c, and fewer AHI events than the total T2D group (DR and no-DR).

**Table 1:** Characteristics of the participants

	HC (*n* = 15)	All T2D (*n* = 62)	T2D no-DR (*n* = 26)	T2D with DR (*n* = 36)	*p*-value (All T2D vs. HC)	*p*-value (DR vs. no-DR)
Age, years, (mean, ±SD)	52.4 (6.7)	54.8 (6.2)	54.2 (6.7)	55.2 (5.9)	0.2233	0.5327
Female, (n, %)	11 (73%)	36 (58%)	15 (58%)	21 (58%)	0.4277	1.0000
BMI, *kg/m*^*2*^, *(median*,* IQR)*	35.9 [27.9, 38.9]	32.2 [28.7, 35.5]	30.3 [28.7, 36.5]	33.0 [30.3, 35.4]	0.5412	0.6126
Current smoking, (n, %)	1 (6.7%)	12 (19.4%)	5 (19.2%)	7 (19.4%)	0.4433	1.0000
Systolic blood pressure, mmHg, (mean, ±SD)	127 (16.1)	132 (16.0)	126 (14.0)	136 (16.3)	0.3189	0.0193*
Diastolic blood pressure, mmHg, (mean, ±SD)	80.2 (10.9)	80.4 (8.2)	78.3 (8.8)	82.0 (7.5)	0.9260	0.0836
Anti-hypertensive use, (n, %)	3 (20%)	50 (80.6%)	21 (80.8%)	29 (80.6%)	< 0.0001**	1.0000
Statin use, (n, %)	1 (6.7%)	50 (80.6%)	19 (73.1%)	31 (86.1%)	< 0.0001**	0.3288
LDL cholesterol, *mg/dL*,* (median*,* IQR)*	110 [82, 125]	93 [70, 120]	97 [76, 118]	85 [70, 120]	0.2110	0.7111
Triglycerides, *mg/dL*,* (median*,* IQR)*	73 [52, 90]	122 [89, 192]	132 [112, 199]	108 [84, 172]	0.0014**	0.1155
Diabetes duration, years, (median, IQR)	NA	11.5 [6.0, 20.0]	6.50 [3.3, 15.3]	15.5 [10.0, 22.0]	NA	0.0011**
Insulin use, (n, %)	NA	32 (52%)	8 (31%)	24 (67%)	NA	0.0113*
HbA1c, *expressed as % and mmol/mol*,* (mean*,* ±SD)*	5.4% (0.3) and 36 (3) *mmol/mol*	8.2% (1.8) and 66 (20) *mmol/mol*	7.8% (1.7) and 62 (14) *mmol/mol*	8.5% (1.8) and 69 (20) *mmol/mol*	< 0.0001**	0.0899
Creatinine, *mg/dL*,* (median*,* IQR)*	0.87 [0.79, 0.96]	0.88 [0.77, 1.12]	0.91 [0.81, 1.20]	0.87 [0.75, 1.07]	0.4300	0.2431
*Urinary aMT6s / creatinine ratio*,* ng/mg*,* (median*,* IQR)*	*11.30 [7.59*,* 21.90]*	*4.06 [1.69*,* 15.10]*	*14.60 [4.26*,* 22.70]*	*1.25 [0.83*,* 4.01]*	0.0489*	0.0007**
*PIPR*,* (median*,* IQR)*	*0.361 [0.259*,* 0.409]*	*0.202 [0.107*,* 0.324]*	*0.295 [0.197*,* 0.352]*	*0.166 [0.089*,* 0.225]*	0.0010**	0.0007**
AHI, *events/hour*,* (median*,* IQR)*	7.9 [2.3, 10.6]	16.3 [6.8, 25.8]	16.9 [5.4, 31.8]	15.2 [7.1, 22.8]	0.0173*	0.9261

All data are expressed as median (interquartile range) or frequency (%) except for age and HbA1c, which are expressed as mean (standard deviation). Asterisks indicate statistical significance (*P < 0.05; **P < 0.01)

*Abbreviations*: HC: Healthy Control, T2D: Type 2 Diabetes Mellitus, DR: Diabetic Retinopathy, n: Number, SD: Standard Deviation, BMI: Body Mass Index, IQR: Interquartile Range, LDL: Low Density Lipoprotein, HbA1c: Hemoglobin A1c, NA: Not Applicable, aMT6s: Nocturnal 6-Sulfatoxymelatonin, PIPR: Post-Illumination Pupillary Light Reflex, AHI: Apnea Hypopnea Index

**Fig. 1:**
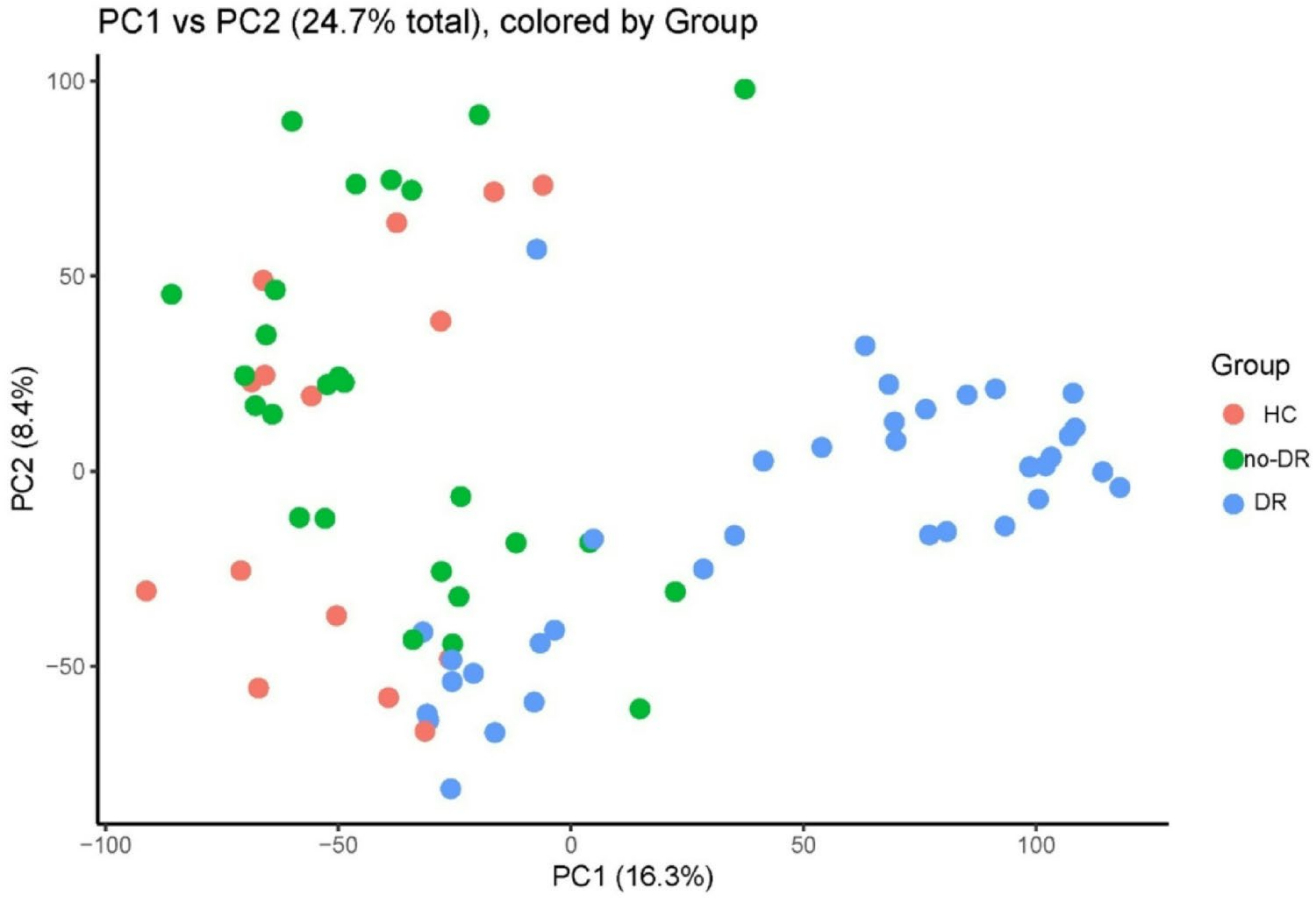
PCA of Metabolomic Profiles. The plot shows separation of serum metabolomic profiles showing partial clustering of DR (blue), no-DR (green), and HC (red) groups. DR samples tend to separate from no-DR and HC, while the latter two largely overlap

PCA showed partial clustering of three groups, with DR samples tending to separate from no-DR and HCs, although some overlap remained (Fig. 1). No-DR and HCs largely overlapped, underscoring that the most pronounced metabolomic differences were between DR and the other groups. *IPA revealed no significantly enriched metabolic pathways*,* and no metabolites were correlated with either PIPR or urinary aMT6s in nominal or adjusted analyses.*

Differential abundance analysis identified 387 metabolites significantly altered between DR and no-DR, while no differences were observed between total T2D and HCs. Filtering for log2 fold change ≥ 1.5 yielded 58 metabolites, with candidate biomarkers summarized in Table 2 and the full list provided in Supplementary Table *S1*. A heatmap of their relative abundances (Supplementary Figure S1) illustrates differences between DR and no-DR. *Comparison of DR versus HCs showed 61 significantly altered metabolites*,* all of which overlapped with differentially abundant metabolites between DR and no-DR (Supplemental Table **S2**).*

**Table 2:** Summary of Log2 fold changes and q-values for candidate metabolites in DR vs. No-DR

Metabolites	Subclass	Log2 Fold Change (DR vs. no-DR)	Q-value (DR vs. no-DR)
Phospholipids			
Phosphatidylcholine (24:1(15Z)/24:1(15Z))	Glycerophosphocholine	4.21	0.0068
Phosphatidylserine (18:0/20:4(8Z,11Z,14Z,17Z))	Glycerophosphoserine	-4.35	0.0392
Fatty Acyls			
5-tetradecenoylcarnitine (14:1n-12)	Acylcarnitine	1.56	0.0068
6-hydroxypentadecanedioic acid (15:0)	Long chain fatty acid	-4.62	0.0142
10,16-dihydroxy-palmitic acid (16:0)	Long chain fatty acid	-3.79	0.0425
Bile acids, Steroids, and Precursors			
Glycochenodeoxycholic acid 7-sulfate	Bile acid	3.23	0.0400
Ganodosterone	Ergostane steroid	-3.80	0.0162
Geranylgeranyl diphosphate	Isoprenoid phosphate	3.19	0.0021
Amino acids			
Isovalerylalanine	N-acyl-L-alpha amino acid	2.85	0.0064
N-(3-Oxododecanoyl) homoserine lactone	Alpha amino acid ester	2.66	0.0076
Environmental Exposures			
Monomethyl phthalate	Benzoic acid ester	3.20	0.0037
Organic Compounds			
1,4-Dihydroxy-2-naphthoic acid	Carboxylic acid	-5.15	0.0036
Butyl ethyl malonate	Dicarboxylic acid	-6.61	0.0189
Diethyl succinate	Fatty acid ester	3.68	0.0313
Pantothenic acid 4’-O-b-D-glucoside	Fatty acyl glycoside	4.93	0.0015
Cobalt-dihydro-precorrin 6	Cobalamin derivative	4.44	0.0045
Biliverdin-IX-alpha	Bilirubin	2.07	0.0419

Findings included higher 5-tetradecenoylcarnitine and phosphatidylcholine (24:1/24:1) and lower long-chain fatty acids (6-hydroxypentadecanedioic acid (15:0) and 10,16-dihydroxy-palmitic acid (16:0)). Monomethyl phthalate, a metabolite linked to plastic exposure and endocrine disruption, was significantly elevated in DR. Two metabolites, 12-HETE and 3,4-DHBA, previously implicated in DR pathogenesis, were also significantly higher, although not above the log2 fold change ≥ 1.5 threshold.

## Discussion

Our findings suggest that DR is associated with metabolic alterations beyond those seen in T2D alone. Unsupervised PCA revealed modest group separation between DR versus no-DR and HCs, supporting global metabolic differences in DR. *Metabolites differentially abundant in DR versus HCs completely overlapped with those identified in DR versus no-DR*,* supporting the robustness of these DR-associated metabolic alterations.*

We observed elevated levels of 5-tetradecenoylcarnitine. Although prior studies have reported mixed findings regarding acylcarnitine levels in DR, our results add to this evolving literature. Concurrent reductions in long-chain fatty acids in DR may reflect broader disruptions in lipid metabolism, though mechanistic interpretations remain speculative. The observed increases in 12-HETE and 3,4-DHBA, help validate our findings, as they are well documented in metabolomic studies and agree with previous literature [2]. Both metabolites have been found to be independent risk markers for DR progression, thus making them possible therapeutic targets [2]. 

Of interest, monomethyl phthalate exhibited significant upregulation in DR versus no-DR *and HCs*, consistent with its potential role as a risk factor for diabetes as reported in meta-analysis [6]. Since monomethyl phthalate, a chemical derived from plastics, has been acknowledged as an endocrine disruptor correlated with increased serum TNFa and decreased adiponectin levels [6], it suggests a link between inflammation and the development of diabetic complications such as DR.

*ipRGC function is known to be lower in DR* [3, 5], *though no significant correlations were observed between metabolites and PIPR*,* suggesting that metabolic alterations seen in DR may not be secondary to ipRGC dysfunction. Similarly*,* negative findings were found between metabolites and urinary aMT6s. These findings are hypothesis-generating and should be interpreted cautiously given study limitations.*

A strength of this study is the inclusion of HCs, which allowed differentiation of metabolic changes specifically associated with DR from those related more generally to T2D. This three-group design offered a broader perspective on disease-specific perturbations. This study is limited by its smaller sample size, cross-sectional nature, and potential residual confounding despite covariate adjustment. Nonetheless, the observed metabolic differences warrant further investigation in larger, prospective cohorts to validate potential biomarkers and clarify future avenues of research.

## Supplementary Information

Below is the link to the electronic supplementary material.

Supplementary Figure S1: Heatmap comparing T2D participants with DR vs. T2D participants with no-DR. The rows representing the selected metabolites were Z-scored across samples and hierarchically clustered using the Euclidean clustering metric. The color key across the top row shows the sample grouping with green representing the no-DR participants and red representing DR participants. The cells in the heatmap were color coded depending on the Z-score with high expression colored red, low expression colored blue, and average expression colored white. Supplementary Material 1

Supplementary Material 2

Supplementary Material 3

## Data Availability

The data that support this study’s findings are not openly available due to participant consent limitations and are available upon reasonable requests to the authors. Data are located in the Research Resources Center (RRC) data portal at the University of Illinois at Chicago, which is a controlled access data storage.

## Declarations

### Trial Registration

Part of the data came from the clinical study, registered at www.clinicaltrials.gov, NCT04547439, date of registration 9/14/2020.
