# Supplementary figures and images for: Exploring metabolomic clues in diabetic retinopathy: a pilot study
Source: Acta Diabetol. 2026 Mar 17;63(6):1137–41. doi: 10.1007/s00592-026-02678-5 (PMC13272203; doi:10.1007/s00592-026-02678-5)

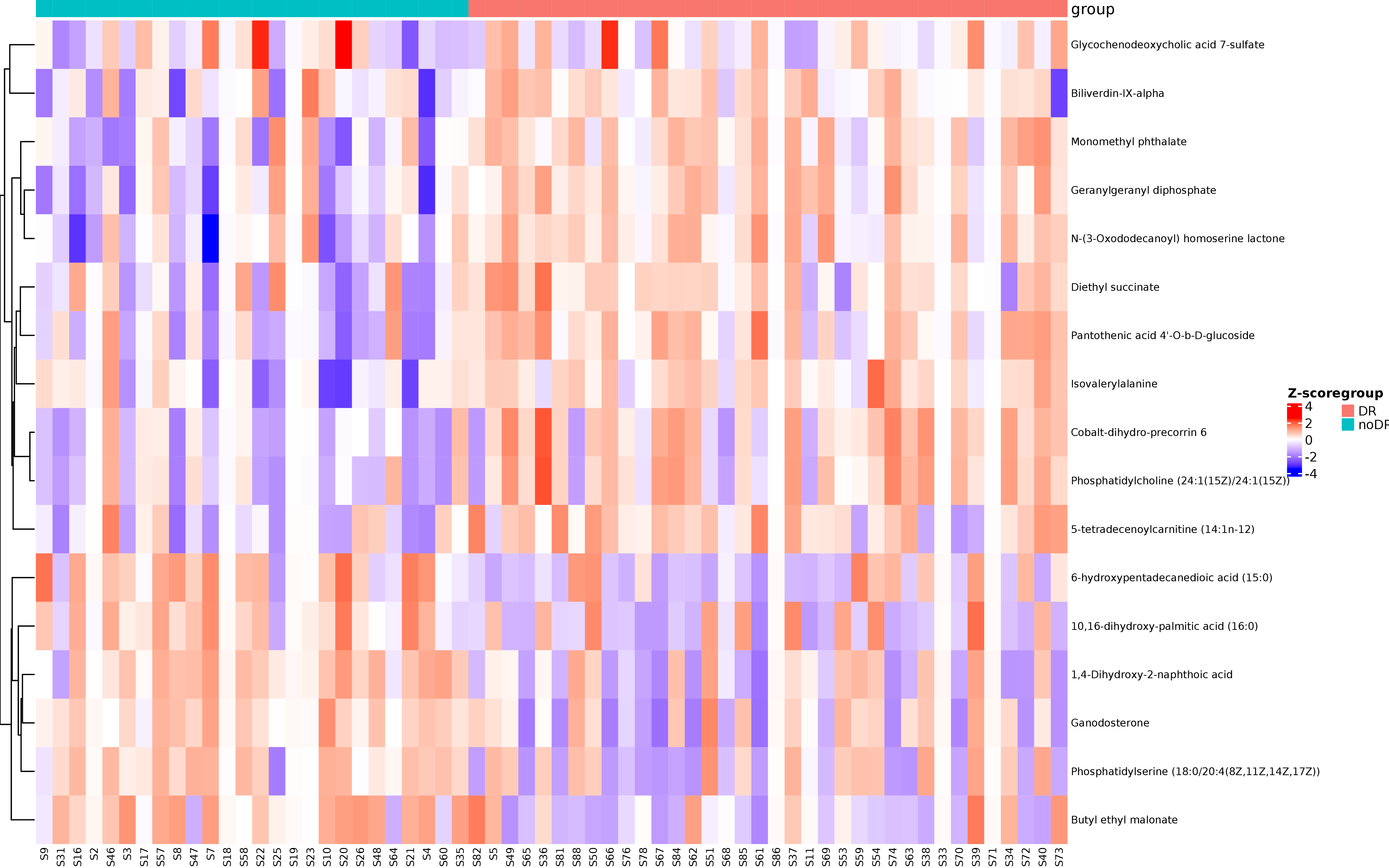

Supplement: Supplementary file 1 — Supplementary Figure S1: Heatmap comparing T2D participants with DR vs. T2D participants with no-DR. The rows representing the selected metabolites were Z-scored across samples and hierarchically clustered using the Euclidean clustering metric. The color key across the top row shows the sample grouping with green representing the no-DR participants and red representing DR participants. The cells in the heatmap were color coded depending on the Z-score with high expression colored red, low expression colored blue, and average expression colored white. Supplementary Material 1 [file 592_2026_2678_MOESM1_ESM.tiff]
