# Supplementary material for: Exploring metabolomic clues in diabetic retinopathy: a pilot study
Source: Acta Diabetol. 2026 Mar 17;63(6):1137–41. doi: 10.1007/s00592-026-02678-5 (PMC13272203; doi:10.1007/s00592-026-02678-5)
Supplement: Supplementary file 2 — Supplementary Material 2 [file 592_2026_2678_MOESM2_ESM.docx]

**Exploring Metabolomic Clues in Diabetic Retinopathy: A Pilot Study**

Matthew Simonson, BS^a^; Yanliang Li, MD/PhD^b^; J. Jason McAnany, PhD^b^; Jason C. Park, PhD^b^; Felix Y. Chau, PhD^b^; Bharati Prasad, MD^c,d^; Silvana Pannain, MD^e^; Erin C. Hanlon, PhD^e^; Eve Van Cauter, PhD^e^; Kirstie K. Danielson, PhD^f^; Brian T. Layden, MD/PhD^f^; Hui Chen, PhD^g^; George E. Chlipala, PhD^h^; Carlos Martinez, PhD^h^; Stephanie J. Crowley, PhD^i^; Sirimon Reutrakul, MD^f^

**Journal:** Acta Diabetologica

**Corresponding author**:

Matthew Simonson - University of Illinois Chicago, 835 S. Wolcott, Suite E625, Chicago, Illinois 60612, Email: [msimon29@uic.edu](mailto:msimon29@uic.edu)

**Supplementary Table S1:** Summary of Log2 fold changes and q-values for all differential metabolites in DR vs. No-DR

| **Metabolites** | **Log2 Fold Change (DR vs. no-DR)** | **Q-value (DR vs. no-DR)** |
| --- | --- | --- |
| Butyl ethyl malonate | -6.61 | 0.0190 |
| 1,4-Dihydroxy-2-naphthoic acid | -5.15 | 0.0037 |
| Triethyl citrate | -5.09 | 0.0058 |
| Verimol C | -4.95 | 0.0018 |
| 3,4,5,6-Tetrahydro-4-hydroxy-6-methyl-2H-pyran-2-one | -4.76 | 0.0325 |
| 6-Hydroxypentadecanedioic acid | -4.62 | 0.0142 |
| Difluprednate | -4.44 | 0.0060 |
| PS (18:0/20:4(8Z,11Z,14Z,17Z)) | -4.30 | 0.0392 |
| Karbutilate | -4.02 | 0.0429 |
| Cincassiol B | -3.98 | 0.0076 |
| Ganodosterone | -3.81 | 0.0163 |
| 10,16-dihydroxy-palmitic acid | -3.79 | 0.0426 |
| 4-Methoxycinnamic acid | -3.63 | 0.0089 |
| Homofukinolide | -3.53 | 0.0307 |
| Methyl 3,4,5-trimethoxycinnamate | -3.51 | 0.0310 |
| Ethyl cinnamate | -3.11 | 0.0146 |
| 6alpha-Fluoro-17-hydroxypregn-4-ene-3,20-dione acetate | -3.00 | 0.0351 |
| Indocyanine green | -2.98 | 0.0177 |
| Diethyl (2R,3R)-2-methyl-3-hydroxysuccinate | -2.78 | 0.0480 |
| Dikegulac | -2.65 | 0.0326 |
| beta-Mangostin | -2.42 | 0.0296 |
| Lansiumamide A | -2.18 | 0.0371 |
| Emedastine | -2.02 | 0.0160 |
| Elenaic acid | -1.95 | 0.0279 |
| Leucyl-Isoleucine | -1.87 | 0.0254 |
| Ethyl Oxalacetate | -1.65 | 0.0302 |
| 5-Tetradecenoylcarnitine | 1.56 | 0.0069 |
| p-Nitrophenyl-O-ethyl ethylphosphonate | 1.80 | 0.0306 |
| Biliverdin-IX | 2.07 | 0.0419 |
| Puromycin | 2.24 | 0.0241 |
| Hexyl glucoside | 2.25 | 0.0043 |
| 3-Hydroxydodecanoic acid | 2.34 | 0.0118 |
| Antrafenine | 2.39 | 0.0388 |
| p-Isopropylphenol | 2.42 | 0.0134 |
| N-(3-Oxododecanoyl)homoserine lactone | 2.66 | 0.0076 |
| Neoacrimarine K | 2.70 | 0.0255 |
| 1-Methoxy-1-pentyloxyethane | 2.81 | 0.0057 |
| Isovalerylalanine | 2.85 | 0.0065 |
| Sulfamoxole | 3.06 | 0.0146 |
| Geranylgeranyl diphosphate | 3.19 | 0.0021 |
| Monomethyl phthalate | 3.20 | 0.0037 |
| Glycochenodeoxycholic acid 7-sulfate | 3.23 | 0.0401 |
| 5-O-Desmethyldonepezil | 3.37 | 0.0058 |
| Propionaldehyde | 3.39 | 0.0216 |
| Euphornin | 3.58 | 0.0440 |
| Salviaflaside methyl ester | 3.58 | 0.0177 |
| Diethyl succinate | 3.68 | 0.0313 |
| Auriculoside | 3.75 | 0.0092 |
| 11-alpha-O-beta-D-Glucopyranosyl-16alpha-O-methylneoquassin | 3.89 | 0.0288 |
| Nonate | 4.07 | 0.0205 |
| Glucosyl (E)-2,6-Dimethyl-2,5-heptadienoate | 4.07 | 0.0369 |
| PC (24:1(15Z)/24:1(15Z)) | 4.21 | 0.0069 |
| Perilloside C | 4.31 | 0.0271 |
| Caryoptosidic acid | 4.34 | 0.0395 |
| Cobalt-dihydro-precorrin 6 | 4.44 | 0.0045 |
| Pantothenic acid 4'-O-b-D-glucoside | 4.93 | 0.0015 |
| Apimaysin | 5.00 | 0.0014 |
| Slaframine | 5.31 | 0.0051 |

**Supplementary Table S2:** Summary of Log2 fold changes and q-values for all differential metabolites in DR vs. HCs

| **Metabolites** | **Log2 Fold Change (DR vs. HCs)** | **Q-value (DR vs. HCs)** |
| --- | --- | --- |
| Butyl ethyl malonate | -5.35 | 0.0047 |
| 3,4,5,6-Tetrahydro-4-hydroxy-6-methyl-2H-pyran-2-one | -5.15 | 0.0198 |
| 1,4-Dihydroxy-2-naphthoic acid | -4.96 | 0.0000 |
| Verimol C | -4.68 | 0.0000 |
| Difluprednate | -4.42 | 0.0000 |
| N-(1-Deoxy-1-fructosyl)methionine | -4.24 | 0.0042 |
| 4-Methoxycinnamic acid | -4.24 | 0.0013 |
| Methyl 3,4,5-trimethoxycinnamate | -4.09 | 0.0004 |
| 3-Hydroxytetradecanedioic acid | -3.97 | 0.0013 |
| Triethyl citrate | -3.92 | 0.0004 |
| Ethyl cinnamate | -3.89 | 0.0057 |
| Gingerdiol 5-acetate | -3.87 | 0.0000 |
| Diethyl (2R,3R)-2-methyl-3-hydroxysuccinate | -3.74 | 0.0161 |
| 6-Hydroxypentadecanedioic acid | -3.68 | 0.0004 |
| Ganodosterone | -3.66 | 0.0039 |
| Litcubinine | -3.57 | 0.0011 |
| Cincassiol B | -3.53 | 0.0001 |
| Homofukinolide | -3.39 | 0.0000 |
| 10,16-dihydroxy-palmitic acid | -3.11 | 0.0004 |
| Diethyl fumarate | -3.00 | 0.0388 |
| S-(Hydroxyphenylacetothiohydroximoyl)-L-cysteine | -2.97 | 0.0001 |
| 6alpha-Fluoro-17-hydroxypregn-4-ene-3,20-dione acetate | -2.96 | 0.0032 |
| Ligballinol | -2.78 | 0.0078 |
| Aspartyl-glutamate | -2.67 | 0.0359 |
| Lansiumamide A | -2.63 | 0.0118 |
| Glutamyl-tryptophan | -2.56 | 0.0125 |
| Furaneol 4-glucoside | -2.46 | 0.0036 |
| Elenaic acid | -2.43 | 0.0061 |
| Diisopropyl adipate | -2.25 | 0.0036 |
| Leucyl-Isoleucine | -2.00 | 0.0167 |
| Diacetoxyscirpenol | -1.99 | 0.0042 |
| Glycerol 1-propanoate diacetate | -1.98 | 0.0037 |
| Phenylalanyl-Histidine | -1.76 | 0.0234 |
| Emedastine | -1.67 | 0.0078 |
| Sterebin B | -1.66 | 0.0149 |
| Geranylgeranyl diphosphate | 1.55 | 0.0093 |
| Biliverdin-IX | 1.58 | 0.0000 |
| Hexyl glucoside | 1.70 | 0.0000 |
| p-Isopropylphenol | 1.70 | 0.0037 |
| Puromycin | 1.75 | 0.0001 |
| Hydroxydodecanoic acid | 1.79 | 0.0072 |
| 1-Methoxy-1-pentyloxyethane | 1.80 | 0.0031 |
| Alanopine | 1.83 | 0.0130 |
| Sulfoglycolithocholate | 1.88 | 0.0000 |
| N-(3-Oxododecanoyl)homoserine lactone | 2.04 | 0.0000 |
| Monomethyl phthalate | 2.22 | 0.0129 |
| Sulfamoxole | 2.22 | 0.0119 |
| Cobalt-dihydro-precorrin 6 | 2.33 | 0.0289 |
| 5-O-Desmethyldonepezil | 2.39 | 0.0016 |
| Lucidenic acid D1 | 2.43 | 0.0000 |
| Auriculoside | 2.49 | 0.0000 |
| PC (24:1(15Z)/24:1(15Z)) | 2.57 | 0.0025 |
| Salviaflaside methyl ester | 2.67 | 0.0036 |
| glycochenodeoxycholic acid 7-sulfate | 2.76 | 0.0033 |
| Nonate | 2.99 | 0.0015 |
| 11-alpha-O-beta-D-Glucopyranosyl-16alpha-O-methylneoquassin | 3.10 | 0.0022 |
| Dibutyl malate | 3.20 | 0.0051 |
| Caryoptosidic acid | 3.47 | 0.0095 |
| Perilloside C | 3.54 | 0.0351 |
| Acidissiminol epoxide | 4.04 | 0.0013 |
| (R)-Pantothenic acid 4'-O-b-D-glucoside | 4.21 | 0.0474 |
