# Supplementary material for: Exploring metabolomic clues in diabetic retinopathy: a pilot study
Source: Acta Diabetol. 2026 Mar 17;63(6):1137–41. doi: 10.1007/s00592-026-02678-5 (PMC13272203; doi:10.1007/s00592-026-02678-5)
Supplement: Supplementary file 3 — Supplementary Material 3 [file 592_2026_2678_MOESM3_ESM.docx]

**Conflict of Interest Disclosure Form**

As the corresponding author, I confirm that I am authorized to complete and sign this disclosure form on behalf of all co-authors. I have collected and reviewed conflict of interest declarations from all authors, and the information provided herein accurately reflects the disclosures of all contributors to this manuscript.

The authors declare that they have no known competing financial interests or personal relationships that could have appeared to influence the work reported in this paper.

The author is an Editorial Board Member/Editor-in-Chief/Associate Editor/Guest Editor for this journal and was not involved in the editorial review or the decision to publish this article.

The authors declare the following financial interests/personal relationships which may be considered as potential competing interests:

S.R. received speaker fees from Eli Lilly. S.P. and served on advisory boards for Novo Nordisk and Eli Lilly. Other authors report no conflicts of interest.

**Signature**:
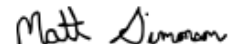
 **Date**: 8/7/2025
